# Passivity based nonlinear model predictive control (PNMPC) of multi-robot systems for space applications

**DOI:** 10.3389/frobt.2023.1181128

**Published:** 2023-06-05

**Authors:** Serdar Kalaycioglu, Anton De Ruiter

**Affiliations:** Department of Aerospace Engineering, Toronto Metropolitan University, Toronto, ON, Canada

**Keywords:** space robotics, model predictive control, multi-robot system, robotic manipulators, passivity

## Abstract

In the past 2 decades, there has been increasing interest in autonomous multi-robot systems for space use. They can assemble space structures and provide services for other space assets. The utmost significance lies in the performance, stability, and robustness of these space operations. By considering system dynamics and constraints, the Model Predictive Control (MPC) framework optimizes performance. Unlike other methods, standard MPC can offer greater robustness due to its receding horizon nature. However, current literature on MPC application to space robotics primarily focuses on linear models, which is not suitable for highly non-linear multi-robot systems. Although Nonlinear MPC (NMPC) shows promise for free-floating space manipulators, current NMPC applications are limited to unconstrained non-linear systems and do not guarantee closed-loop stability. This paper introduces a novel approach to NMPC using the concept of passivity to multi-robot systems for space applications. By utilizing a passivity-based state constraint and a terminal storage function, the proposed PNMPC scheme ensures closed-loop stability and a superior performance. Therefore, this approach offers an alternative method to the control Lyapunov function for control of non-linear multi-robot space systems and applications, as stability and passivity exhibit a close relationship. Finally, this paper demonstrates that the benefits of passivity-based concepts and NMPC can be combined into a single NMPC scheme that maintains the advantages of each, including closed-loop stability through passivity and good performance through one-line optimization in NMPC.

## 1 Introduction

During the last 2 decades, there has been a significant interest in free-flying autonomous multi-robot systems for space applications (Nanos and Papadopoulos, 2017). Such an autonomous robotics system may be employed to assemble large space structures (e.g., communications antennae, and telescopes), manufacture on-orbit as well as to provide services for other space assets (i.e., exchange of orbital removal units (ORU), refuelling of spacecrafts, removing space debris, etc.). Until recently, some of these operations (e.g., inspection, exchange of ORUs) have been carried out by astronauts as Extravehicular Activities (EVA) in low Earth orbit. However, extensive astronauts training, operations planning and preparations, the associated cost, schedule and time delays and inherent risky nature of space environment for astronauts convinced space agencies to look into other more efficient alternatives such as autonomous robotics spacecrafts (Papadopoulos et al., 2021). Furthermore, in high Earth orbit or other unmanned space exploration missions this may be the only option available. One of the pioneers in demonstrating robotics spacecraft capabilities was the Engineering Test Satellite ETS-VII of JAXA and it consisted of chasing and capturing spacecrafts (Yoshida, 2003). The ARCHINAUT, which is developed by NASA and Made in Space, features multiple robotic arms that are designed to perform on-orbit assembly and precision manufacturing. Other systems that are capable of performing similar tasks include the Kontur, METERON, and SPIDERFAB. The Gateway Moon Space Station is currently being developed by NASA and other international organizations. Other projects that are currently in development include the Moon Village and the Mars exploration mission of the European Space Agency (ESA) and OrbitalHub by the German Space Agency (DLR) (Orbital Hub DLR Vision, 2022). NASA and MAXAR Technologies Inc. are currently developing a robotic spacecraft known as OSAM-1 (NASA’s Robotic OSAM-1 Mission, 2022; Krebs D., 2022). It will be able to perform various tasks on-orbit including the assembly of satellites, debris removal and the refueling of them. In 2016, China’s Aolong-1 satellite was launched and demonstrated the capabilities of space robotic vehicles by capturing and handling space debris. Through a robotic arm, the satellite was able to grasp and send the object it collected back on a re-entry trajectory. Small-scale robotic systems, exemplified by the Future Space Debris Removal Orbital Manipulator (FSDROM), are poised to make a significant contribution to upcoming space debris removal missions, according to Shyam et al. (2021). As reported by the European Space Agency (ESA) in 2019, the amount of space debris in Earth’s orbit has exceeded 8,000 tons and continues to rise, posing a significant threat to both satellites and astronauts (Chatterjee, 2014). Consequently, the removal of space debris has emerged as a top priority in space missions (Space Foundation White Paper, 2020). Direct capture of objects is one approach for mitigating the issue, and it can be executed through rigid or flexible capture methods, as classified by Zhao et al., 2020. A variety of techniques for flexible direct capture, including harpoons, nets, and tentacles, have been suggested by Billot et al., 2014; Zhang and Huang, 2016; Forshaw et al., 2017. Such mechanisms for capture help mitigate the risk of collisions between debris and space robots, thereby minimizing the likelihood of failed capture attempts that could result in further debris generation, as stated by Biesbroek et al., 2017.

Space manipulators often encounter unidentifiable, rotating debris, leading to harm to both the robot’s structure and its actuators. Thus, it is essential to implement a controlling law that is robust enough to sustain its performance in the event of actuator malfunction or failure. This circumstance is particularly probable when employing direct capture methods since the capture process can lead to significant effects on the spacecraft, as stated by Seweryn et al., 2022.

However, there are major technological challenges in autonomous robotics spacecrafts. First of all, missions may involve execution of uncertain tasks in an unstructured environment. There may be inherent uncertainties in the system (e.g., friction, geometry, stiffness, and damping, etc.) and payload parameters (e.g., mass/inertia, geometry, momentum, etc.) (Aghili, 2020). The conventional model-based controllers are not robust to deal with these uncertainties on orbit. Ground testing as well as on-orbit characterization and evaluations are difficult and proved to be limited. Furthermore, the contact models and payload characteristics cannot be determined in advance when dealing with unknown debris, etc. Moreover, the actuators are subject to saturation and the controller must deal with these constraints. In addition, there may be actuator or sensor failures and the system has to work with limited degrees of freedom and sensory feedback in case of occurrence of faults (Raisi et al., 2022).

Therefore, conventional controllers cannot cope with these types of tasks, system and payload uncertainties, unknown payload and contact dynamics behaviour, constraints such as problem of force/torque saturation, and partial system failures such as faulty actuator or sensor. Recently, a new type of control system known as Model Predictive Control (MPC) has gained widespread attention from industry and academia (Hewing et al., 2020). There are also variations of MPC techniques such as implicit, explicit, adaptive, gain-scheduled, and non-linear and others (Rawlings, 2000; Christofides et al., 2013; Morato et al., 2020; Fin, 2021; Shi and Zhang, 2021).

Achieving optimal performance, stability, and robustness is crucial in space operations. The Model Predictive Control (MPC) framework addresses this challenge by considering system dynamics and constraints during performance optimization. Its receding horizon nature also makes it more robust than other methods (Shuyou et al., 2014). However, current literature on MPC application to space robotics mainly focuses on linear models, which are not suitable for highly non-linear multi-robot systems commonly found in space applications.

Despite its numerous advantages, Model Predictive Control (MPC) presents several challenges that require further investigation, including feasibility, nonlinearity, closed-loop stability, and robustness (Rybus et al., 2018; Psomiadis and Papadopoulos, 2022). When the models of the plant or the constraints are nonlinear, Nonlinear Model Predictive Control (NMPC) schemes must be employed (Shuyou et al., 2014; Quirynen et al., 2015; Vukov et al., 2015; Wang et al., 2016; Englert et al., 2019; Rathai, 2020; Kalaycioglu and de Ruiter, 2023). However, it has been observed in (Jadbabaie et al., 1999) that NMPC does not always ensure closed-loop stability. Additionally, the issue of robustness arises in MPC when model uncertainty or noise is present, as is often the case due to the inability of the prediction model to precisely match the actual dynamics of the plant being controlled (Nanos and Papadopoulos, 2011).

NMPC presents a promising solution for free-floating space manipulators. However, existing NMPC applications are limited to unconstrained non-linear systems and cannot guarantee closed-loop stability. Passivity theory is a potent instrument for examining and managing nonlinear systems, as demonstrated by numerous works by Wang. and Xie, 2014; Schaft 2000; Raff et al. (2007) introduced a significant advancement in this field through the proposal of a Nonlinear Model Predictive Control (NMPC) scheme based on passivity. This development was motivated by the interdependencies between optimal control, passivity, and NMPC. This scheme incorporates particular passivity-based constraints that ensure both closed-loop stability and feasibility.

To address this gap in space robotics, this paper proposes a novel approach to NMPC that uses the concept of passivity for multi-robot systems specifically designed for space-related applications. The proposed NMPC scheme utilizes a passivity-based state constraint to ensure closed-loop stability. Thus, this approach provides an alternative to the use of control Lyapunov function for controlling nonlinear multi-robot space systems and related applications. This is because stability and passivity are highly interrelated. The passivity based NMPC scheme offers a robust solution for optimizing performance while maintaining stability and ensuring system robustness in space robotics.

The main contribution of this paper lies in the application of the concept of passivity-based NMPC to multi-robot systems for space applications. While the idea of passivity-based NMPC has been previously explored by Raff et al. (2007), their work only involved a simple simulation case concerning a quadruple tank system. Wang. and Xie, 2014 proposed a passivity-based attitude control approach for rigid bodies that can be used in spacecraft and satellite applications. In this paper, the authors have developed a novel passivity-based state constraint and a terminal storage function specifically applicable to the nonlinear dynamics of multi-robotic systems. The developed terminal storage function ensures asymptotic closed-loop stability and superior performance. It is important to note that while nonlinear MPC (NMPC) has shown promise for a single free-floating space manipulator system (as demonstrated in the works of Papadopoulos et al., 2021; Rybus et al., 2018), current NMPC applications do not guarantee closed-loop stability if the full nonlinear dynamics terms are retained in the controller. Moreover, it is worth noting that the majority of the studies in this area have implemented NMPC for a single planar space arm with two or three degrees of freedom.

Therefore, the paper addresses the full nonlinear dynamics while guaranteeing closed-loop stability for a multi-robot system. The approach presents a significant advancement in the field of NMPC for multi-robot systems in space applications, thanks to the novel passivity-based state constraint and terminal storage function specifically designed for multi-robotic systems.

A proper choice for the proposed control algorithm’s hardware and software implementation for spacecraft robotics system would be a distributed processor architecture, which provides parallel control of the spacecraft and robots, reduced computer speed demands, and inherent redundancy for enhanced reliability. The proposed architecture may include three main processors: 1) Spacecraft Attitude Determination and Control System (ADACS) and Robot Control System (RCS), 2) Command and Data Handling (C&DH), and 3) Communications Processor. The proposed control algorithm can be housed in the ADACS and RCS computer, which controls the spacecraft’s attitude, orbital maneuvers, and robots’ end-effector position and orientation. Sensory data from sources like star trackers, joint encoders, and vision systems can also be collected. The Computer Software Configuration Items (CSCIs) may include modules for OCA, the proposed PNMPC, Forward and Inverse Kinematics Functions, Robot Dynamics Function, Jacobian, Jacobian Rate, and more, all of which can be located in the ADACS and RCS computer.

This paper is organized as follows: Section 2 provides the equations of motions for the compounded multi-robot spacecraft system. Section 3 introduces the concept of passivity and the proposed passivity based NMPC algorithm for a free-flying multi-robot space system. In Section 4, the computer simulation results, their analysis and discussion are presented. Section 5 outlines the conclusions and suggestions for future work.

## 2 System dynamics

**FIGURE 1:**
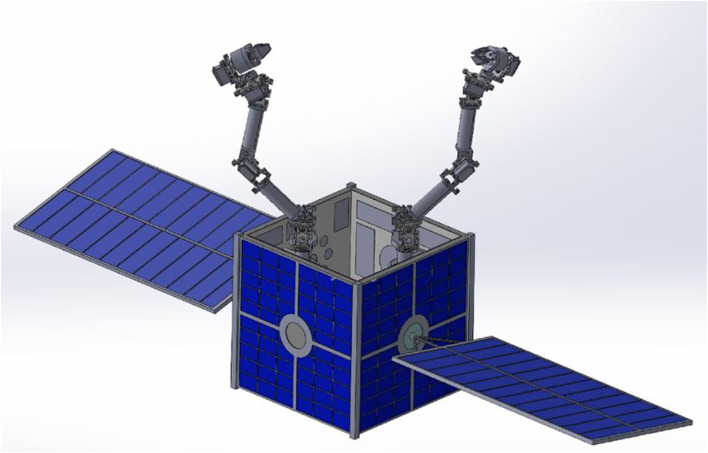
Multi-robot spacecraft system concept.

The system being studied comprises of a chaser spacecraft, a target payload, and two redundant manipulators with *n* degrees of freedom. An illustration of a similar system with a chaser spacecraft, two *n*-degree robots, and a rigid payload can be seen in Figure 1.

The set of equations that govern the motions of two robots and a chaser spacecraft can be expressed as:

$$G\;\ddot{\tilde{q}}\left(t\right)+D\;\dot{\tilde{q}}\left(t\right)=\tilde{\Gamma }$$ (1)

$${\ddot{\tilde{q}}}^{T}=\left[{\ddot{\tilde{R}}}_{c},{\dot{\tilde{\omega }}}_{c},{\ddot{\tilde{\theta }}}_{L},{\ddot{\tilde{\theta }}}_{R}\right]$$ (1a)

$${\tilde{\Gamma }}^{T}=\left[{\tilde{F}}_{c},{\tilde{\tau }}_{c},{\tilde{\tau }}_{\theta_{L}},{\tilde{\tau }}_{\theta_{R}}\right]$$ (1b)

where ${\ddot{\tilde{q}}}^{T}$ is a vector containing the linear and angular acceleration of the chaser spacecraft and the joint angular accelerations for the left and right arm, and ${\tilde{\Gamma }}^{T}$ is a vector containing the external control force and moments for the chaser spacecraft and the joint control torques for the left and right arm. The matrix G is a positive definite matrix that represents the mass/inertia, while D is a matrix that includes non-linear terms such as Coriolis and centrifugal terms. The linear and angular acceleration of the chaser spacecraft are represented by ${\dot{\tilde{R}}}_{c}$ , ${\dot{\tilde{\omega }}}_{c}$ , respectively. ${\ddot{\tilde{\theta }}}_{L}$ , ${\ddot{\tilde{\theta }}}_{R}$ are the joint angular accelerations for the left and right arm respectively, while ${\tilde{F}}_{c},{\tilde{\tau }}_{c}$ are the external control force and moments for the chaser spacecraft. The joint control torques for the left and right arm are represented by ${\tilde{\tau }}_{\theta_{L}}$ $,{\tilde{\tau }}_{\theta_{R}}$ , respectively.

To enhance the mathematical expression of the dynamics equations, it is possible to represent them in the Cartesian domain for the robots and in terms of Euler angles for the spacecraft. This can be achieved by utilizing the Jacobian of the system, denoted as $\underline{J}$ , which facilitates the transformations from joint space to Cartesian space for the robots and from the body-fixed angular rates to Euler rates for the spacecraft. The resulting equations can provide a more insightful understanding of the system dynamics and can be particularly useful for control and optimization purposes:

$$G^{*}\ddot{\tilde{\mu }}\left(t\right)+D^{*}\dot{\tilde{\mu }}\left(t\right)=\tilde{F}$$ (2)

where

$${\tilde{\mu }}^{T}\left(t\right)=\left[{\tilde{R}}_{c},\varphi ,\psi ,\theta ,{\tilde{X}}_{L},{\tilde{X}}_{R}\right]$$ (2a)

${\tilde{R}}_{c}$ , the position vector of the center of mass of the spacecraft, the rotational transformation from orbital to body axes is represented by a 1-3-2 Euler sequence, with angles φ, ψ and θ, respectively and ${\tilde{X}}_{L},{\tilde{X}}_{R}$ are the pose vectors for the left and the right end-effectors, respectively. By utilizing the Pseudo-inverse Jacobian matrix $J^{*}$ for the redundant system, one can calculate the equivalent mass/inertia matrix $G^{*}$ , Coriolis matrix $D^{*}$ , and the force vector $\tilde{F}$ in the Cartesian domain for the compounded multi-robot spacecraft system.

$$\dot{\tilde{\mu }}\left(t\right)=J\dot{\tilde{q}}\left(t\right)$$ (3)

$$J^{*}=(J^{T}J){}^{-1}\;J^{T}$$ (4)

$$G^{*}={J^{*}}^{T}G\;J^{*}$$ (5)

$$D^{*}=\left({J^{*}}^{T}\;D\;J^{*}-{J^{*}}^{T}G\;J^{*}\;\dot{J}\;J^{*}\right)$$ (6)

$$\tilde{F}={J^{*}}^{T}\;\tilde{\Gamma }$$ (7)

It can be shown that ${\dot{G}}^{*}-2D^{*}$ is a skew-symmetric matrix in the following form and this relationship can be used in developing an appropriate storage function in Section 3.

$${\dot{G}}^{*}-2D^{*}=-{J^{*}}^{T}G\;J^{*}\dot{J}J^{*}-2\;{J^{*}}^{T}D\;J^{*}+{J^{*}}^{T}\;{\dot{J}}^{T}G\;{J^{*}}^{T}J^{*}$$ (8)

Furthermore, the non-linear equations of the system dynamics can be written as:

$${\tilde{y}}^{T}\left(t\right)=\left[{\tilde{\mu }}^{T}\left(t\right),\dot{\tilde{\mu }}\left(t\right)\right]$$ (9a)

$$\begin{array}{c} \dot{\tilde{y}}\left(t\right)=\tilde{g}\left(\tilde{y}\right)+f\left(\tilde{y}\right)\tilde{u}\left(t\right)\\ \tilde{z}\left(t\right)=\tilde{h}\left(\tilde{y}\right) \end{array}$$ (9b)

$$\tilde{g}\left(\tilde{y}\right)=\left[\begin{array}{cc} 0 & 1\\ 0 & -{G^{*}}^{-1}D^{*} \end{array}\right]\tilde{y}\left(t\right)\;f\left(\tilde{y}\right)=\left[\begin{array}{c} 0\\ {G^{*}}^{-1} \end{array}\right]$$

$$\tilde{h}\left(\tilde{y}\right)=C\tilde{y}\left(t\right)\;\tilde{y}\left(0\right)=\tilde{y}\left(t_{0}\right)$$ (9c)

where the control input function is denoted by $\tilde{u}\left(t\right)$ , while $\tilde{z}\left(t\right)$ represents the output and C is the observation matrix The non-linear system function representing the system dynamics is denoted by $\tilde{g}\left(\tilde{y}\right)$ while $f\left(\tilde{y}\right)$ is the input coefficient function, and $\tilde{h}\left(\tilde{y}\right)$ is the non-linear function used to obtain the output vector based on the state variables.

## 3 The concept of passivity and passivity based NMPC

A dynamics system can be classified as passive if there is a storage function $V\left({\tilde{y}}_{t}\right)$ that meets the following condition:

$$V\left({\tilde{y}}_{t}\right)-V\left({\tilde{y}}_{0}\right)\leq \int_{t_{0}}^{t}{\tilde{u}}^{T}\left(\tau \right)\tilde{z}\left(\tau \right)\;d\tau$$ (10a)

Furthermore, a formal characterization of a system as Input Feed-forward Output Feedback Passive (IF-OFP) is established when a storage function $V\left({\tilde{y}}_{t}\right)$ exists that satisfies the certain conditions:

$$V\left({\tilde{y}}_{t}\right)-V\left({\tilde{y}}_{0}\right)\leq \int_{t_{0}}^{t}\left[{\tilde{u}}^{T}\left(\tau \right)\tilde{z}\left(\tau \right)-\rho \;{\tilde{z}\left(\tau \right)}^{T}\tilde{z}\left(\tau \right)-\upsilon \;{\tilde{u}}^{T}\left(\tau \right)\tilde{u}\left(\tau \right)\right]\;d\tau$$ (10b)

The positive passivity indices, denoted by ρ and υ , characterize the passivity properties of the system under consideration.

**FIGURE 2:**
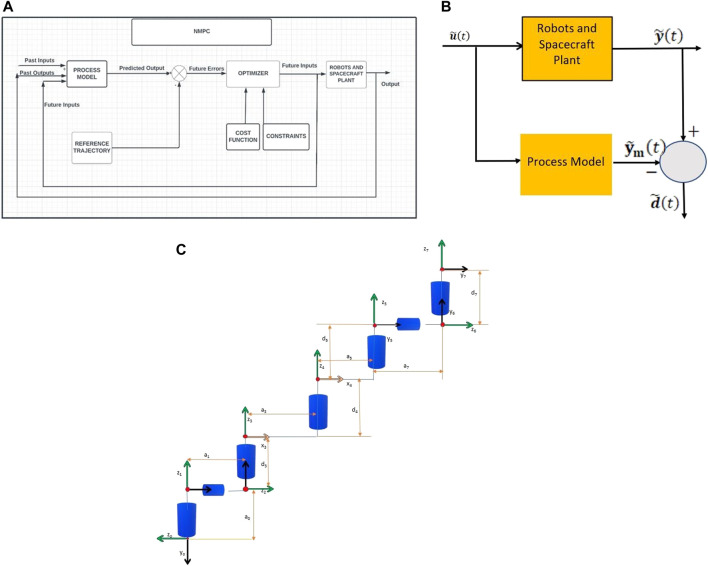
**(A)** NMPC Block diagram. **(B)** Bias computation with an independent model. **(C)** Robot geometry used in the simulations.

Raff et al. (2007) have established that the passivity-based nonlinear model predictive control scheme can achieve local asymptotic stability of a system, provided the system is passive and possesses a continuously differentiable storage function $V\left({\tilde{y}}_{t}\right).$ The conventional NMPC formulation for the dual arm nonlinear system can be written as:

$$\min\;\frac{1}{2}\int_{0}^{T_{p}}\left[{\left(\tilde{y}\left(t\right)–\;{\tilde{y}}_{r}\left(t\right)\right)}^{T}\;K\;\left(\tilde{y}\left(t\right)–\;{\tilde{y}}_{r}\left(t\right)\right)+{\tilde{u}}^{T}\left(t\right)\;W\;\tilde{u}\left(t\right)\right]\;dt$$ (11)

subject to Eq 9a, Eq 9b, Eq 9c and where $T_{p}$ is the prediction horizon; K and W are positive definite square weighting matrices and ${\tilde{y}}_{r}\left(t\right)$ is the reference trajectory Nonlinear model predictive control (NMPC) is founded on the principle of iteratively solving the finite horizon optimal control problem in real-time to determine the optimal control input. Figure 2A depicts the NMPC block diagram. Schaft 2000 and Jadbabaie et al. (1999) highlighted that while NMPC can be useful, it does not always guarantee closed-loop stability.

Furthermore, the plant output $\tilde{y}\left(t\right)$ might be different than the model output ${\tilde{y}}_{m}\left(t\right)$ in the presence of model parameter uncertainties. To overcome model parameter uncertainties in the process, both the model and the plant are simulated under the same control system input as shown in Figure 2B. The first output is the real plant output, represented as $\tilde{y}\left(t\right)$ while the second output is the model output, represented as ${\tilde{y}}_{m}\left(t\right)$ .

The calculations are performed within the control block diagram’s “ROBOTS AND SPACECRAFT PLANT” and “PROCESS MODEL”, as illustrated in Figure 2A. Subsequently, the bias $\tilde{d}\left(t\right)$ is determined, using the following equation:

$$\tilde{d}\left(t\right)=\tilde{y}\left(t\right)-{\tilde{y}}_{m}\left(t\right)$$ (12)

The process predictions are then calculated from model predictions by adding a bias correction term to each prediction over the horizon. The details of these calculations are provided as part of Eq. 22c when a set of discretized system equations is employed.

The Passivity-Based Nonlinear Model Predictive Control (PNMPC) scheme can be organized in the following manner through the incorporation of a terminal cost as the storage function $V\left({\tilde{y}}_{T_{p}}\right)$ to guarantee the closed loop stability (Mayne et al., 2000; Raff et al., 2007):

$$\min\;V\left({\tilde{y}}_{T_{p}}\right)+\frac{1}{2}\int_{0}^{T_{p}}\left[{\left(\tilde{y}\left(t\right)–\;{\tilde{y}}_{r}\left(t\right)\right)}^{T}\;K\left(\tilde{y}\left(t\right)–\;{\tilde{y}}_{r}\left(t\right)\right)+{\tilde{u}}^{T}\left(t\right)\;W\;\tilde{u}\left(t\right)\right]\;dt$$ (13)

subject to Eq. 9a and the following inequality constraints:

$$\frac{dV\left({\tilde{y}}_{t}\right)}{dt}\leq {\tilde{u}}^{T}\left(t\right)\;\tilde{z}\left(t\right)\;where\;\tilde{z}\left(t\right)=\dot{\tilde{\mu }}\left(t\right)$$ (14)

$${\tilde{u}}^{T}\left(t\right)\;\tilde{z}\left(t\right)\leq -\rho \;{\tilde{z}\left(t\right)}^{T}\tilde{z}\left(t\right)-\upsilon \;{\tilde{u}}^{T}\left(t\right)\tilde{u}\left(t\right)\;\rho \geq 0,\upsilon \geq 0$$ (15)

To establish the passivity constraint, the initial step is to define the tracking error vector, which represents the deviation between the output and the reference trajectory as follows:

$${\tilde{e}}_{y}=\tilde{\mu }\left(t\right)-{\tilde{\mu }}_{r}\left(t\right)$$ (16)

In order to attain proficient tracking performance, one may contemplate utilizing the following storage function in Cartesian domain as proposed in this paper.

$$V\left({\tilde{y}}_{t}\right)=\frac{1}{2}\;\left({\dot{\tilde{\mu }}}^{T}\left(t\right)\;G^{*}\;\dot{\tilde{\mu }}\left(t\right)+{{\tilde{e}}_{y}}^{\;T}R{\tilde{e}}_{y}\right)$$ (17)

where $\underline{R}$ is a positive definite, symmetrical square matrix, and subsequently calculate the time derivative of $V\left({\tilde{y}}_{t+T_{p}}\right)$ to obtain the following constraint equations.

$$\frac{d\;V\left({\tilde{y}}_{t}\right)}{dt}=\frac{d}{dt}\;\left[\frac{1}{2}\;\left({\dot{\tilde{\mu }}}^{T}\left(t\right)\;G^{*}\;\dot{\tilde{\mu }}\left(t\right)+{{\tilde{e}}_{y}}^{\;T}R\;{\tilde{e}}_{y}\right)\right]$$ (18)

Please note that ${\dot{G}}^{*}-2D^{*}$ is a skew-symmetric matrix and enables the following simplification which was used in calculating the time derivative of the storage function $V\left({\tilde{y}}_{t}\right)$ in Eq. 18.

$${\left[\dot{\tilde{\mu }}\right.}^{T}\left(t\right)\;\left({\dot{G}}^{*}-2D^{*}\right)\;\dot{\tilde{\mu }}\left(t\right)]=0$$ (19)

The time derivative of the proposed storage function can now be calculated using Eq. 18 as shown below:

$$\begin{array}{ll} \frac{dV\left({\tilde{y}}_{t}\right)}{dt} & =1/2\left\{\ddot{\tilde{\mu }}{\left(t\right)}^{T}G^{*}\dot{\tilde{\mu }}\left(t\right)+\dot{\tilde{\mu }}{\left(t\right)}^{T}G^{*}\ddot{\tilde{\mu }}\left(t\right)+\dot{\tilde{\mu }}{\left(t\right)}^{T}\;{\dot{G}}^{*}\dot{\tilde{\mu }}\left(t\right)+{\dot{\tilde{e}}}_{y}^{T}R\;{\tilde{e}}_{y}\right.\\ & \;\left.+\;{{\tilde{e}}_{y}}^{T}R\;{\dot{\tilde{e}}}_{y}\right\} \end{array}$$

$$\;\;=\dot{\tilde{\mu }}{\left(t\right)}^{T}G^{*}\ddot{\tilde{\mu }}\left(t\right)+\dot{\tilde{\mu }}{\left(t\right)}^{T}\;{\dot{G}}^{*}\dot{\tilde{\mu }}\left(t\right)+{\dot{\tilde{e}}}_{y}^{T}R\;{\tilde{e}}_{y}$$ (20a)

Please note that both $G^{*}$ and R matrices are symmetrical. Further simplification of Eq. 20a can be achieved by adding and subtracting $2D^{*}$ into equation and taking advantage of the skew-symmetry property represented in Eq. 19. The resulting equation is

$$\frac{dV\left({\tilde{y}}_{t}\right)}{dt}=\dot{\tilde{\mu }}{\left(t\right)}^{T}G^{*}\ddot{\tilde{\mu }}\left(t\right)+\frac{1}{2}\;\left\{\dot{\tilde{\mu }}{\left(t\right)}^{T}\left(\dot{G^{*}}-2D^{*}+2D^{*}\right)\;\dot{\tilde{\mu }}\left(t\right)\right\}+{\dot{\tilde{e}}}_{y}^{T}\;R\;{\tilde{e}}_{y}$$ (20b)

By utilizing Eq. 19, it is possible to simplify Eq. 20b and which can then be written in a more concise form as follows.

$$\frac{dV\left({\tilde{y}}_{t}\right)}{dt}=\dot{\tilde{\mu }}{\left(t\right)}^{T}G^{*}\ddot{\tilde{\mu }}\left(t\right)+\left\{\dot{\tilde{\mu }}{\left(t\right)}^{T}D^{*}\;\dot{\tilde{\mu }}\left(t\right)\right\}+{\dot{\tilde{e}}}_{y}^{T}\;R\;{\tilde{e}}_{y}$$ (20c)

where ${\dot{\tilde{e}}}_{y}=\dot{\tilde{\mu }}\left(t\right)-{\dot{\tilde{\mu }}}_{r}\left(t\right)\;.$

$$\frac{dV\left({\tilde{y}}_{t}\right)}{dt}=\dot{\tilde{\mu }}{\left(t\right)}^{T}\;\left\{G^{*}\ddot{\tilde{\mu }}\left(t\right)+D^{*}\dot{\tilde{\mu }}\left(t\right)+R{\tilde{e}}_{y}\right\}$$ (20d)

By utilizing Eq. 2, the aforementioned expression can be reformulated as follows:

$$\frac{dV\left({\tilde{y}}_{t}\right)}{dt}=\dot{\tilde{\mu }}{\left(t\right)}^{T}\left\{\tilde{F}+R\;{\tilde{e}}_{y}\right\}={\left(\tilde{F}+R\;{\tilde{e}}_{y}\right)}^{T}\;\tilde{z}\left(t\right)$$ (20e)

Thus, the following terminal value of the predicted control input proposed in Eq. 21a satisfies the equality in Eq. 20e as well as guarantees the passivity-based inequality constraint in (Eq. 14).

$$\tilde{u}\;\left(t\right)=\tilde{u}\;\left(T_{p}\right)=\left(\tilde{F}+R\;{\tilde{e}}_{y}\right)\;t=T_{p}$$ (21a)

Hence, it is hereby proven that the proposed terminal value of predicted control input and the storage function satisfy the constraint provided in Eq. 14 as follows:

$$\frac{\left.dV\left({\tilde{y}}_{T_{p}}\right)\right)}{dt}={\left(\tilde{F}+R\;{\tilde{e}}_{y}\right)}^{T}\;\dot{\tilde{\mu }}\backslash \left(t\right)={\tilde{u}}^{T}\left(t\right)\dot{\tilde{\mu }}\left(t\right)\leq {\tilde{u}}^{T}\left(t\right)\;\tilde{z}\left(t\right)\;t=T_{p}$$ (21b)

where the value of $\tilde{F}$ is calculated by utilizing the model-based computed torque/force approach at *t =* $T_{p}.$ It is part of the terminal value of the predicted input and not measured from the plant output as illustrated in Figure 2A. The first inequality constraint provided in (Eq. 14) is now satisfied by the proposed storage function $V\left({\tilde{y}}_{t}\right)$ (Eq. 17) and the proposed control input (Eq. 21a). In order to enforce the closed loop stability, the other passivity constraints provided in Eq. 15 are implemented as part of the NMPC model. i.e.

$$\begin{array}{c} {\tilde{u}}^{T}\left(t\right)\;\dot{\tilde{\mu }}\left(t\right)\leq -\rho {\dot{\tilde{\mu }}}^{T}\left(t\right)\;\dot{\tilde{\mu }}\left(t\right)-\upsilon \;{\tilde{u}}^{T}\left(t\right)\;\tilde{u}\left(t\right)\\ \rho \geq 0,\upsilon \geq 0 \end{array}$$ (21c)

The following prediction equations are employed in conjunction with the system discrete state-space model at each sampling instant $k_{t}$ (Kalaycioglu, S and de Ruiter A. 2023). These equations are the discretized version of Eq. 9b. and written as follows:

$$\Delta \tilde{y}\left(k_{t}+j+1\right)=\underline{\hat{A}}\left(\hat{g}\left(k_{t}+j\right)\right)\Delta \tilde{y}\left(k_{t}+j\right)+\underline{\hat{B}}\left(\hat{g}\left(k_{t}+j\right)\right)\;\Delta \tilde{u}\left(k_{t}+j\right)$$

$$\Delta \tilde{y}\left(k_{t}+j\right)=\tilde{y}\left(k_{t}+j\right)-{\tilde{y}}_{r}\left(k_{t}+j\right)$$

$$\Delta \tilde{u}\left(k_{t}+j\right)=\tilde{u}\left(k_{t}+j\right)-\tilde{u}\left(k_{t}+N_{p}\right)$$

$$j=1\ldots N_{p}$$ (22a)

Where $\tilde{u}\left(k_{t}+N_{p}\right)$ is the terminal value of the predicted input at t = $T_{p.}\;\left(i.e.,\tilde{F}+R\;{\tilde{e}}_{y}\right).$ These prediction equations are then substituted into the discretized version of the cost function (Eq. 13) and the optimum predicted control inputs $\Delta \tilde{u}\left(k_{t}+j\right)$ are obtained as follows:

$$\Delta \tilde{u}\left(k+j\right)={-\left({\underline{\hat{B}}}^{T}\;\underline{K}\;\underline{\hat{B}}+\underline{W}\right)}^{-1}{\underline{\hat{A}}}^{T}\underline{K}\;\underline{\hat{B}}\;\left(\tilde{y}\left(k_{t}+j\right)-{\tilde{y}}_{r}\left(k_{t}+j\right)\right)$$

$$Q={\left({\underline{\hat{B}}}^{T}\;\underline{K}\underline{\hat{B}}+\underline{W}\right)}^{-1}{\underline{\hat{A}}}^{T}\underline{K}\;\underline{\hat{B}}$$

$$\Delta \tilde{u}\left(k+j\right)=-Q\;\left(\tilde{y}\left(k_{t}+j\right)-{\tilde{y}}_{r}\left(k_{t}+j\right)\right)$$

$$\tilde{u}\left(k+j\right)=-Q\left(\tilde{y}\left(k_{t}+j\right)-{\tilde{y}}_{r}\left(k_{t}+j\right)\right)+\tilde{u}\left(k_{t}+N_{p}\right)$$ (22b)

Therefore, the passivity based NMPC predictive control yields a state feedback mechanism $\left\{-Q\;\left(\tilde{y}\left(k_{t}+j\right)-{\tilde{y}}_{r}\left(k_{t}+j\right)\right)\right\}$ augmented with a feedforward component $\left\{\tilde{u}\left(k_{t}+N_{p}\right)\right\}$ predicated on the terminal value of the inputs (i.e. $\tilde{F}+R\;{\tilde{e}}_{y})$ . The incorporation of the terminal cost as the storage function $V\left({\tilde{y}}_{T_{p}}\right)$ guarantees the closed loop stability. The mathematical formulations present a novel approach to NMPC for multi-robot systems in space applications by incorporating the full non-linear system dynamics equations in the Cartesian domain using the concept of passivity.

Please also note that the process predictions are calculated from model predictions by adding a bias correction term to each prediction over the horizon *j =1 … Np* to include model parameter uncertainties as part of Eq. 22a when using a discretized set of system equations at the sampling time of $k_{t}$ . where

$$\tilde{y}\left(k_{t}+j\right)={\tilde{y}}_{m}\left(k_{t}+j\right)+\tilde{d}\left(k_{t}\right)$$ (22c)

The proposed PNMPC scheme utilizes a novel passivity-based state constraint and a terminal storage function to ensure closed-loop stability and superior performance. The approach demonstrates that passivity-based concepts and NMPC can be combined into a single scheme that maintains the advantages of each, including closed-loop stability through passivity and good performance through one-line optimization in NMPC. This approach also provides an alternative method to the control Lyapunov function for controlling non-linear multi-robot space systems and applications, as stability and passivity exhibit a close relationship.

## 4 Result and discussion

This section presents and examines the outcomes of computer simulations. Three sets of simulation results are presented, which correspond to 1) the proposed Passivity-Based Nonlinear Model Predictive Control (PNMPC) approach with a storage terminal function and the passivity constraints; 2) PNMPC scheme operating without utilizing a storage terminal function, instead relying solely on passivity constraints and finally 3) a conventional NMPC that lacks the passivity conditions and the storage function in its cost function. The simulation results of these three sets are compared, analyzed, and discussed in detail.

### 4.1 Simulation results—Passivity-based nonlinear model predictive control (PNMPC)

In this section, the proposed passivity-based nonlinear model predictive scheme is applied to control a multi-robot spacecraft. In the simulation, the reference trajectory is characterized by a high level of intricacy. This intricate and complex trajectory serves as a demonstration of the effectiveness of the proposed PNMPC algorithm in applications that demand precise pursuit of intricate and rapid trajectories.

The end-effector is required to track the following trajectory on a plane where the payload is located:

$$\begin{array}{c} x_{r}=x_{0}+a\;\sin\left(\omega_{n}t\right)+a\;\sin\left(\omega_{p}t\right)\\ y_{r}=y_{0}+a\;\sin\left(\omega_{n}t+\phi_{0}\right)+a\;\sin\left(\omega_{p}t+\phi_{0}\right) \end{array}$$

$$z_{r}=z_{o}$$ (23)

where $\left[x_{0},y_{0},{z_{o},\omega }_{n},\omega_{p},\phi_{0},a\right]=\left[0.4,0.25,0.5,2,12,1.6,0.02\right]$ also the initial conditions for the end-effector of the left robot are given as: $\left[x,y,z,{\dot{x}}_{d},{\dot{y}}_{d},{\dot{z}}_{d}\right]=\left[0.6,0.3,0.5,0,0,0\right]$ . The reference trajectory represents co-centric circles. Tables 1, 2 provides the system parameters used in the computer simulations for the two robots and the spacecrafts. Figure 2C illustrates the robot geometry used in the simulation.

**TABLE 1:** System parameters.

Hardware configuration item	Mass (kg)	Dimensions (m) (prism)
Spacecraft	40	0.5 × 0.5 × 0.3
Common Payload	10	0.4 × 1 × 0.4
Joint/Link *1*	1	0.1 × 0.1 × 0.1
Joint/Link 2	1	0.1 × 0.1 × 0.1
Link 3	3	0.43 × 0.1 × 0.1
Link 4	5	0.64 × 0.1 × 0.1
Joint/Link 5	3	0.1 × 0. 1 × 0.1
Joint/Link 6	1	0.1 × 0.1 × 0.1
Joint/Link 7	3	1 × 0.1 × 0.1

**TABLE 2:** Inertia parameters.

	S/C	Payload	Link1	Link2	Link3	Link4	Link5	Link6	Link7
M(kg)	40	10	1	1	3	5	3	1	3
I_xx_ (g-m⧵2)	1,130	830	1.7	1.7	5	8.3	5	1.7	1.7
I_yy_ (g-m⧵2)	1,130	830	1.7	1.7	48.7	174.8	5	1.7	1.7
I_zz_ (g-m⧵2)	1,660	830	1.7	1.7	48.7	174.8	5	1.7	1.7

### 4.2 Part 1: The proposed PNMPC with the storage terminal function and the passivity constraints

In this section, the following cost function provided in Eq. 13, subject to Eq. 14 and Eq. 15 is minimized.

$$\min\;V\left({\tilde{y}}_{T_{p}}\right)+\frac{1}{2}\int_{0}^{T_{p}}\left[{\left(\tilde{y}\left(t\right)–\;{\tilde{y}}_{r}\left(t\right)\right)}^{T}K\left(\tilde{y}\left(t\right)–\;{\tilde{y}}_{r}\left(t\right)\right)+{\tilde{u}}^{T}\left(t\right)\;W\tilde{u}\left(t\right)\right]\;dt$$

**FIGURE 3:**
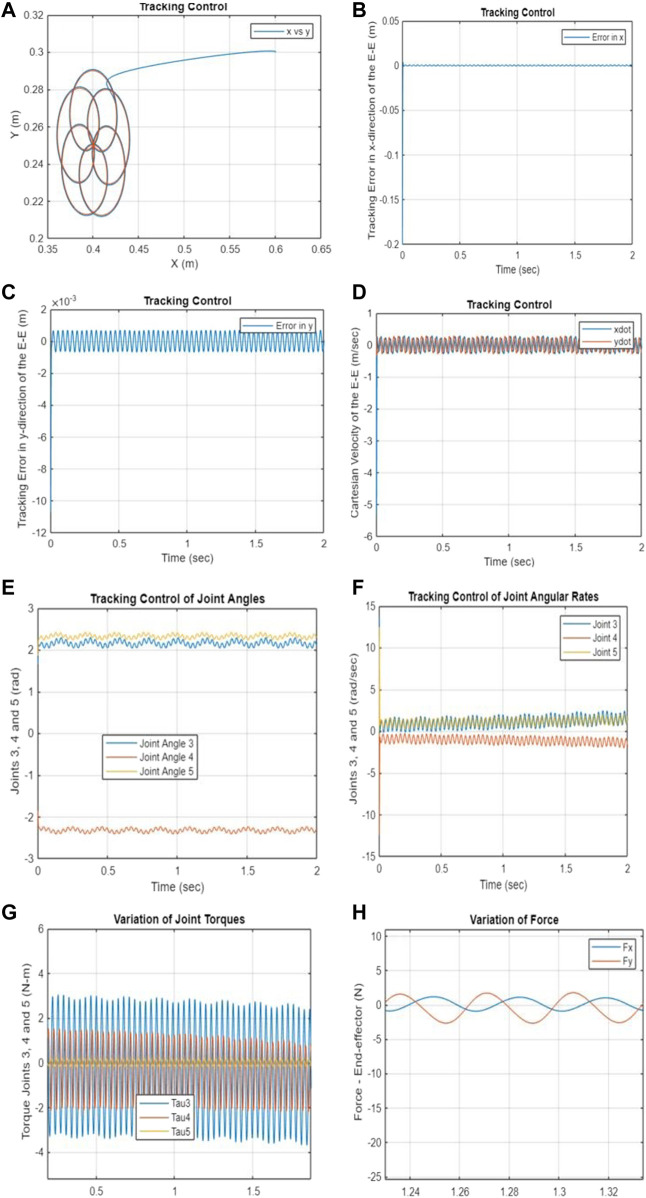
**(A–H)** Tracking performance–PNMPC with a storage function and passivity constraints. **(A)** Cartesian position of the end-effector. **(B)** Tracking error in the x-direction. **(C)** Tracking error in the y-direction. **(D)** Cartesian velocity of the end-effector. **(E)** Tracking control of joints 3–5. **(F)** Tracking control of angular rates for joints 3–5.

Figures 3A–H exhibit the simulation results, which indicate that the developed PNMPC algorithm is remarkably adept at tracking the intricate trajectory. Figure 3A displays the Cartesian position of the end-effector, while Figures 3B,C illustrate the error in Cartesian displacement of the end-effector. Figure 3D illustrates the time variation of the Cartesian velocity of the end-effector. Moreover, Figures 3E–H demonstrate the variations in joint angles over time, angular rates, joint torque, and the equivalent force values, correspondingly.

### 4.3 Part 2: PNMPC1 scheme without the storage terminal function

**FIGURE 4:**
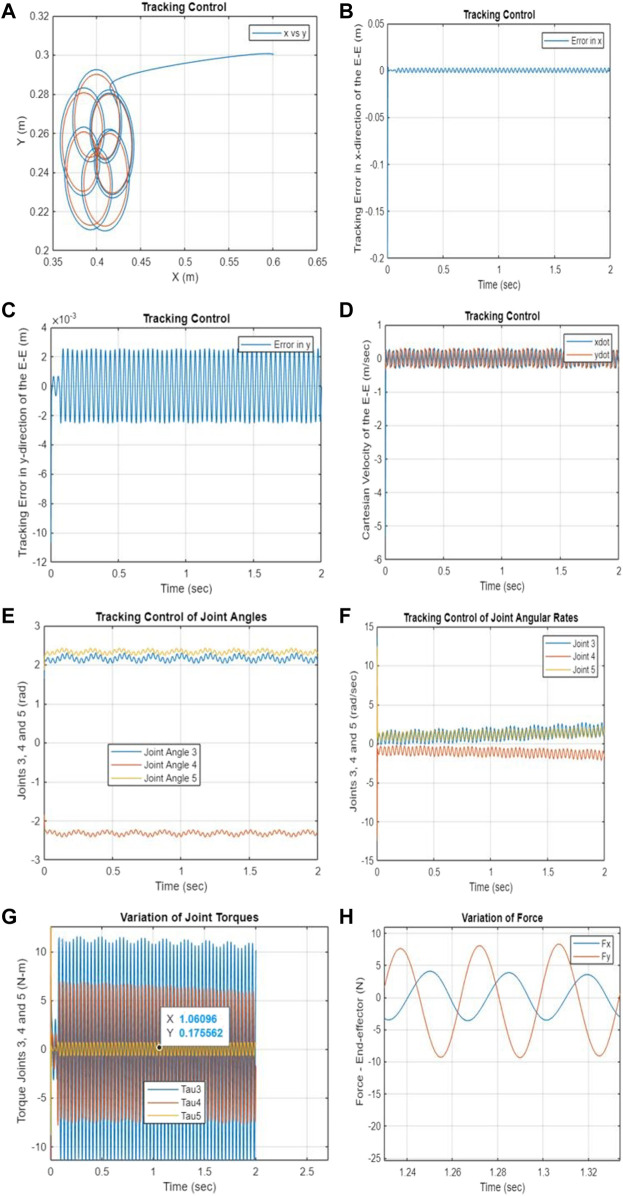
**(A–H)** Tracking performance–PNMPC1 without a terminal function only with passivity constraints. **(A)** Cartesian position of the end-effector. **(B)** Tracking error in the x-direction. **(C)** Tracking error in the y-direction. **(D)** Cartesian velocity of the end-effector. **(E)** Tracking control of joints 3–5. **(F)** Tracking control of angular rates for joints 3–5. **(G)** Variation of torques for joints 3–5. **(H)** Variation of end-effector contact force.

The following PNMPC1 scheme operates without utilizing a storage terminal function, instead relying solely on passivity constraints. The simulation results presented in this section, the storage function $V\left({\tilde{y}}_{t}\right)$ .is removed from the NMPC performance index as a terminal function. Figures 4A–H illustrate the effect of the storage function $V\left({\tilde{y}}_{t}\right)$ . on the tracking performance and stability. The same reference trajectory and the initial conditions are used to compare the simulation results.

By satisfying both Eq. 14 and Eq.15, the simulation results are generated through the minimization of the following cost function subject to negative feedback control law.

$$\min\;\frac{1}{2}\int_{0}^{T_{p}}\left[{\left(\tilde{y}\left(t\right)–\;{\tilde{y}}_{r}\left(t\right)\right)}^{T}K\left(\tilde{y}\left(t\right)–\;{\tilde{y}}_{r}\left(t\right)\right)+{\tilde{u}}^{T}\left(t\right)W\tilde{u}\left(t\right)\right]dt$$

$$\tilde{u}\;\left(t\right)=-Q\;{\tilde{e}}_{y}$$ (24)

**FIGURE 5:**
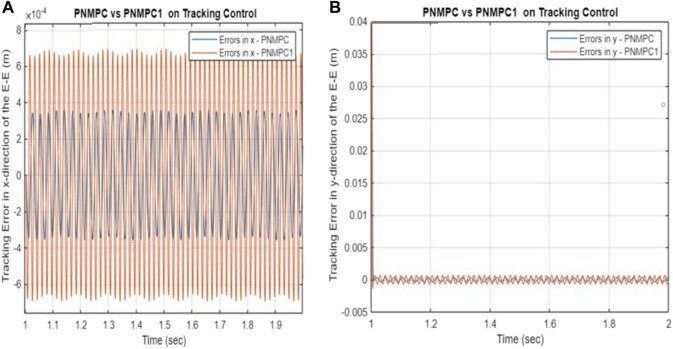
**(A–B)** The impact of the terminal/storage function in the NMPC performance index. **(A)** Comparison of tracking performances in the x-direction between PNMPC and PNMPC1. **(B)** Comparison of tracking performances in the y-direction between PNMPC and PNMPC1.

Based on the results shown in Figures 3A–G and Figures 4A–G, it is evident that the proposed Passivity-based Nonlinear Model Predictive Control (PNMPC) outperforms the PNMPC system that lacks a terminal function (PNMPC1), while still adhering to passivity constraints. While both systems demonstrate stability, the PNMPC approach with the proposed terminal storage function provides notably superior tracking performance. Figures 5A,B display the tracking errors for both algorithms, highlighting the influence of the terminal function in the cost function (or performance index) of the developed NMPC scheme.

### 4.4 Part 3: NMPC scheme without the storage terminal function and passivity constraints

**FIGURE 6:**
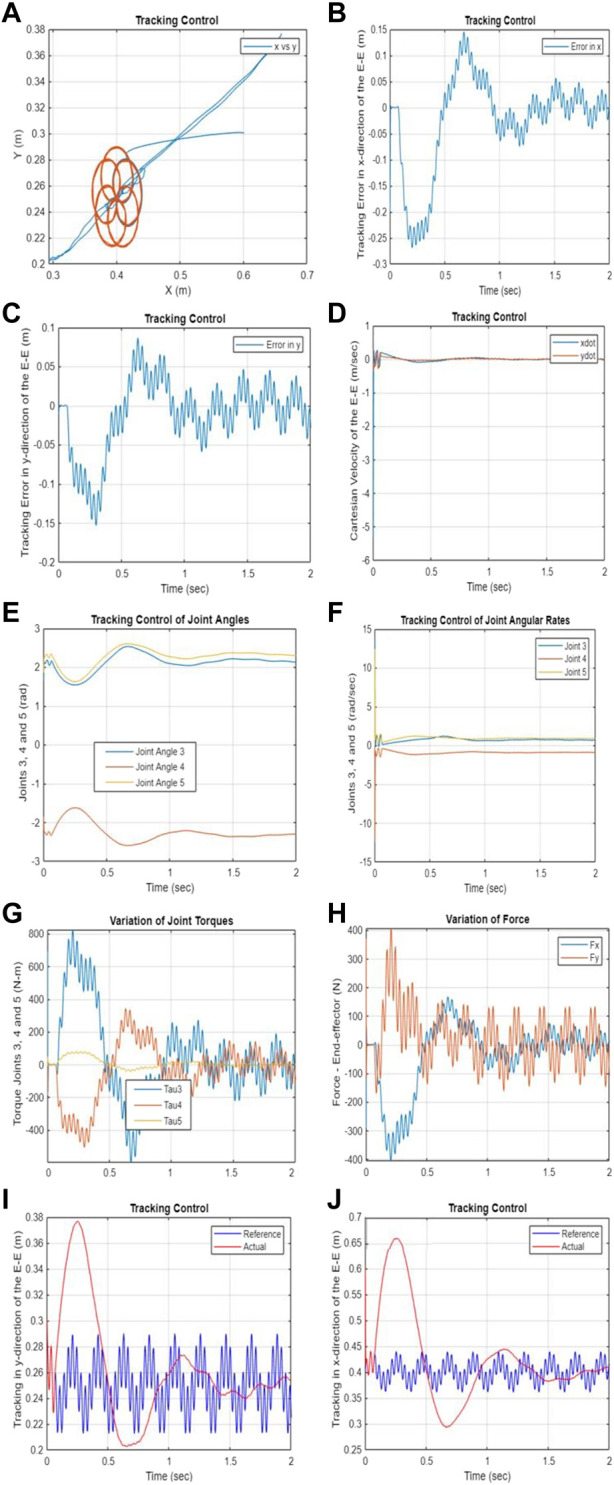
**(A–J)** Tracking performance–PNMPC without a terminal function only with passivity constraints. **(A)** Cartesian position of the end-effector. **(B)** Tracking error in the x-direction. **(C)** Tracking error in the y-direction. **(D)** Cartesian velocity of the end-effector. **(E)** Tracking control of joints 3–5. **(F)** Tracking control of angular rates for joints 3–5. **(G)** Variation of torques for joints 3–5. **(H)** Variation of end-effector contact force. **(I)** Tracking performance in the y-direction– PNMPC without a terminal function only with passivity constraints. **(J)** Tracking performance in the x-direction– PNMPC without a terminal function only with passivity constraints.

In this section, the following cost function provided in Eq. 24 without being subject to Eq. 14 and Eq. 15 is minimized subject to negative feedback control law. Figures 6A–J demonstrate the impact of the absence of both the storage function and passivity constraints on tracking performance and stability. The simulation was conducted using identical reference trajectory and initial conditions to ensure a fair comparison of the results.

The presented Figures 6A–J indicate that the Nonlinear Model Predictive Control (NMPC) algorithm is unable to track the reference trajectory when both the storage function and passivity constraints are absent. The tracking errors are deemed unacceptable, and the results are catastrophic.

### 4.5 Part 4: Summary comparisons of tracking errors for three sets of simulations

**FIGURE 7:**
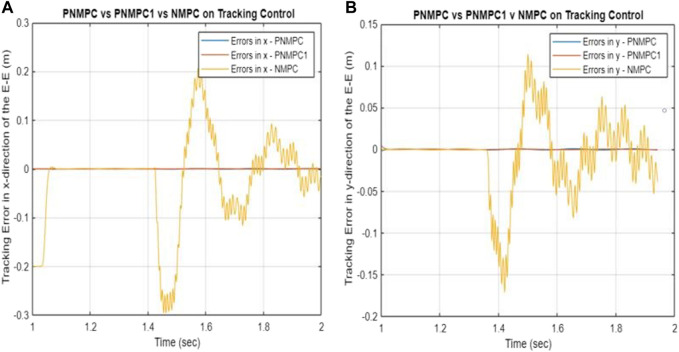
Summary of tracking performances in the **(A)** x-direction and **(B)** y-direction among three algorithms.

Based on the simulation results illustrated in Figures 7A,B, it is evident that the PNMPC system proposed in this study surpasses both the PNMPC system that lacks a terminal function (PNMPC1), while still adhering to passivity constraints, and the regular NMPC scheme (NMPC) that lacks a storage terminal function and is not subject to passivity constraints. While the passivity constraints contribute to stability, the proposed PNMPC approach with the terminal storage function provides notably superior tracking performance. Figures 7A,B display the tracking errors for all three algorithms, emphasizing the impact of the terminal function in the cost function (or performance index) and the presence of passivity constraints in the Nonlinear Model Predictive Control (NMPC) schemes.

## 5 Conclusion and future work

This paper introduced a nonlinear model predictive control method predicated on the concept of passivity for multi-robot systems in space applications. The study and the simulation results demonstrated that utilizing specific passivity-based state constraints, along with a terminal storage function, guaranteed closed-loop stability and resulted in superior tracking performance in simulation results. The proposed approach integrated passivity, optimal control, and nonlinear model predictive control to cater to spacecraft-mounted multi-robot systems, specifically in the context of space applications. Due to the close relationship between stability and passivity, the proposed passivity-based nonlinear model predictive control scheme provided an alternative approach to the control Lyapunov function-based method for redundant nonlinear space robotics systems. The approach was applied to a free-flying spacecraft-based multi-robot system. This paper demonstrated that the benefits of passivity-based concepts and NMPC can be combined into a single NMPC scheme that maintains the advantages of each, including closed-loop stability through passivity and good performance through one-line optimization in NMPC. However, the current study has identified several limitations of the developed PNMPC scheme, which we intend to address in future research. One potential drawback is that inaccuracies in the modeling process could adversely affect its performance. Another limitation is that the PNMPC requires offline tuning of its parameters. Furthermore, the lack of experimental testing in the current study highlights a need for further research to validate the simulation results.

## Data Availability

The original contributions presented in the study are included in the article/Supplementary Material, further inquiries can be directed to the corresponding author.
